# Phosphorylated Mnk1 and eIF4E Are Associated with Lymph Node Metastasis and Poor Prognosis of Nasopharyngeal Carcinoma

**DOI:** 10.1371/journal.pone.0089220

**Published:** 2014-02-14

**Authors:** Jun Zheng, Jiao Li, Lina Xu, Guiyuan Xie, Qiuyuan Wen, Jiadi Luo, Duo Li, Donghai Huang, Songqing Fan

**Affiliations:** 1 Department of Pathology, the Second Xiangya Hospital, Central South University, Changsha, Hunan, China; 2 Department of Oncology, the Second Xiangya Hospital, Central South University, Changsha, Hunan, China; 3 Department of Otorhinolaryngology, the Xiangya Hospital, Central South University, Changsha, Hunan, China; 4 Department of Biochemistry of Changzhi Medical College, Changzhi, Shanxi, China; Columbia University, United States of America

## Abstract

Nasopharyngeal carcinoma (NPC) is a head and neck malignant tumor rare throughout most of the world but common in Southeast Asia, especially in Southern China. The phosphorylation of eukaryotic translation initiation factor 4E (eIF4E) by MAP kinase-interacting kinases (Mnk) on Ser-209 promotes cellular proliferation, survival, malignant transformation and metastasis. However, whether the alterations of the expression of p-eIF4E and p-Mnk1 protein are associated with clinicopathologic/prognostic implication for NPC has not been reported. The purposes of the present study are to examine the expression of p-eIF4E and p-Mnk1 protein in NPC and non-cancerous nasopharyngeal epithelial tissues by immunohistochemistry and evaluate the association between the expression of p-eIF4E and p-Mnk1 protein and clinicopathological characteristics of NPC. The results showed that the positive percentage of p-Mnk1 and p-eIF4E proteins expression in NPC (83.5% and 75.4%, respectively) was significantly higher than that in non-cancerous nasopharyngeal epithelium (40.0% and 32.9%, respectively). The positive expression of p-eIF4E and p-Mnk1 in the NPC with cervical lymph node metastasis was significantly higher than those without lymph node metastasis. Additionally, p-eIF4E expression was more pronouncedly increased in metastatic NPC than the matched primary NPC. Increase of p-eIF4E and p-Mnk1 expression was significantly correlated inversely with overall survival. Spearman’s rank correlation test further showed that expression of p-Mnk1 was strongly positive correlated with expression of p-eIF4E in NPC. The expression of p-Mnk1 and p-eIF4E in NPC was proved to be the independent prognostic factors regardless of lymph node metastasis, clinical stages and combination of radiotherapy and chemotherapy, histological type, age and gender by multivariate analysis. Taken together, high expression of p-Mnk1 and p-eIF4E might be novel valuable biomarkers to predict poor prognosis of NPC and therapeutic targets for developing the valid treatment strategies.

## Introduction

Nasopharyngeal carcinoma (NPC) is a head and neck malignant tumor rare throughout most of the world but common in Southeast Asia, especially in Southern China [1]. Epstein-Barr virus (EBV), environmental factors, and genetic susceptibility play important roles in the pathogenesis of NPC pathogenesis, the EBV in particular has been implicated in the molecular abnormalities leading to NPC [1]–[4]. The molecular pathogenesis of NPC includes abnormal expression and alteration of dominant oncogenes and recessive oncogenes/tumor-suppressor genes and alterations in signaling pathways such as the Akt pathway, mitogen-activated protein kinases, and the Wnt signaling pathway [2]. Therefore, further elucidation of the molecular mechanism underlying NPC is essential for the development of new effective therapeutic agents.

Eukaryotic translation initiation factor 4E (eIF4E) plays a critical role in initiating translation of mRNAs, and up-regulating the expression of tumor relevant proteins, which are involved in activation of proto-oncogenes, angiogenesis, autocrine growth stimulation, cell survival, invasion and communication with the extracellular environment [8]–[13]. Overexpression of eIF4E has been found in many types of tumors and cancer cell lines, but not in typical benign lesions [14]–[20]. Phosphorylation of eIF4E is catalyzed by the MAPK-activated protein kinase called MAP kinase-interacting kinases (Mnks), particularly Mnk1 specifically phosphorylate eIF4E at Ser209 which is the only phosphorylation site in eIF4E. Mnk and eIF4E interact with eIF4G bringing them into physical proximity to facilitate eIF4E phosphorylation [21]–[23]. The eIF4E phosphorylation is the molecular basis of carcinogenesis. Overexpression and/or increased phosphorylation of eIF4E, now considered to be a proto-oncogene, leads to overexpression of certain proto-oncogenes, growth factors, and other cell cycle–related protein transcripts, which promotes proliferation and survival rate of tumor cell and effectively regulates cellular transformation and metastasis [9], [20], [24], [26]–[27]. Some studies have shown that p-eIF4E and p-Mnk1 were respectively correlated with human carcinogenesis and development, and the inhibition of the Mnk1/eIF4E pathway acted as a potential therapeutic target [9]–[10], [13], [24], [27]–[31].

Previous studies have confirmed that there is an overexpression of eIF4E in head and neck tumor including of NPC, and eIF4E can enhance NPC cell proliferation and cell cycle progression [15], [33]. However, whether the alterations of the expression of p-eIF4E and p-Mnk1 protein are associated with development and progression and clinicopathologic/prognostic implication for NPC has not been reported. In the current study, we have investigated the expression pattern of p-eIF4E and p-Mnk1 protein in 272 NPC cases and 85 non-cancerous nasopharyngeal epithelial specimens by Immunohistochemistry (IHC) and determined the correlation between the expression of p-eIF4E and p-Mnk1 and clinicopathologic/prognostic characteristics in NPC. We found that higher expression of p-Mnk1 and p-eIF4E is associated with the cervical lymph node metastasis in NPC. The significantly positive correlation between p-Mnk1 and p-eIF4E expression indicates that eIF4E activation through the Mnk1 plays an important role in the progression of NPC. Moreover, high expression of p-eIF4E in addition to p-Mnk1 might predict poor prognosis of NPC.

## Materials and Methods

### Ethics Statement

Samples were obtained with informed consent and all protocols were approved by the Second Xiangya Hospital of Central South University Ethics Review Board (Scientific and Research Ethics *Committee*, no. s02/2000). Written informed consent was obtained from all patients also the written informed consent was obtained from the next of kin, caretakers, or guardians on the behalf of the minors/children participants involved in your study.

### Tissue Samples and Clinical Data

Two hundred and seventy-two (272) paraffin-embedded NPC cases from the primary NPC patients with their age ranging from 17 to 83 years (median, 48.0 years), also 85 non-cancerous nasopharyngeal epithelial specimens from independent patients with chronic inflammation of nasopharyngeal mucosa, were obtained from the Second Xiangya Hospital of Central South University (Changsha, China). No patient had previously been treated with radiotherapy and chemotherapy at the time of original biopsy. Among these samples, 52 cases had the matched relapse or metastatic cancer specimens besides primary NPC tissues. Complete clinical record and follow-up data were available for all patients. Written informed consent was obtained from all patients, and this study was approved by the Ethics Review Committee of the Second Xiangya Hospital of Central South University. All specimens had been confirmed pathological diagnosis according to WHO histological classification of the NPC. The patients were staged according to the 1997 to the UICC/AJCC staging system of NPC.

The NPC histological patterns and clinical T stages were classified as follows: 247 cases of undifferentiated carcinomas and 25 cases of differentiated non-keratinizing carcinomas; 8 cases of clinical stage I, 86 cases of stage II, 117 cases of stage III, and 61cases of stage IV. Among these patients included in the study, 194 patients were positive for cervical lymph node metastasis and 78 patients were negative. Among 272 patients, 142 patients were treated by radiotherapy alone, 12 patients by chemotherapy alone, and 118 patients by combined radiotherapy and chemotherapy. Complete clinical record and follow-up data of all patients were available. Overall survival time was calculated from the data of diagnosis to the date of death or the data last known alive. A total of 177 patients from (65.1%) were alive with a mean follow-up period of 44.4 months (10–125 months).

### IHC and Scores

The IHC staining for p-Mnk1 or p-eIF4E protein in NPC sections was carried out using ready-to-use Envision ™+Dual Link System-HRP methods (Dako; Carpinteria, CA). The staining conditions for each antibody were adjusted according to our laboratory experience [31]. Briefly, each NPC section was deparaffinized and rehydrated, and high-temperature antigen retrieval was achieved for all antibodies by heating the samples in 0.01 M citrate buffer in a domestic microwave oven at full power (750 Watts) for 15 minutes, then the samples were immersed into methanol containing 0.3% H_2_O_2_ to inactivate endogenous peroxidase at 37°C for 30 minutes. To eliminate nonspecific staining, the slides were incubated with appropriate preimmune serum for 30 minutes at room temperature. After incubation with a 1∶500 dilution of primary antibody to p-eIF4E protein (Rabbit polyclonal, Catalog : #2227-1, Epitomics, Inc.) and with a 1∶500 dilution of primary antibody to p-Mnk1 protein (Rabbit polyclonal IgG p-Mnk1(Thr197/202,Catalog : #2111,Cell Signaling) at 4°C overnight, slides were rinsed with phosphate-buffered saline (PBS) and incubated with a labelled polymer-HRP was added according to the manufacturer’s instructions and incubated 30 minutes. Color reaction was developed by using 3-amino-9-ethylcarbazole (AEC) chromogen solution. All slides were counterstained with hematoxylin. Positive control slides were included in every experiment in addition to the internal positive controls. The specificity of the antibody was determined with matched IgG isotype antibody as a negative control. Moreover, a single band with correct molecular weight in western blotting was identified in our previous published paper [31].

Two pathologists who were blinded to the IHC scores separately carried out the data analysis as described previously [31]. The p-eIF4E and p-Mnk1 staining were scored as negative (<10% staining) and positive (≥10% staining), respectively.

### Statistical Analyses

All statistical analyses were performed using SPSS 13.0. The χ2 test was used to analyze the relationship between the expression of p-eIF4E and p-Mnk1 and clinicopathological characteristics of NPC. The Spearman’s rank correlation coefficient was used to assess the significance of the relation between p-eIF4E and p-Mnk1 expression in NPC. Kaplan-Meier analysis was performed for overall survival curves and statistical significance was assessed using the log-rank test. Overall survival was defined as the time from the treatment initiation (diagnosis) to the date of death. To evaluate whether expression of p-eIF4E and p-Mnk1 are independent prognostic factors of overall survival, multivariate analysis using the Cox proportional hazard regression model was performed. All p values were based on the two-sided statistical analysis and p-value less than 0.05 was considered to be statistically significant.

## Results

### Association between Expression of p-Mnk1 and p-eIF4E Protein and the Clinicopathological Features of NPC

**Figure 1:**
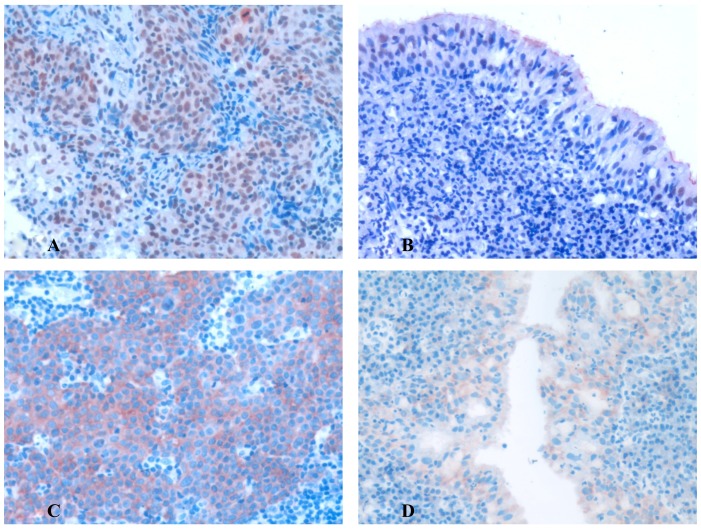
Expression of p-Mnk1 and p-eIF4E protein in NPC and non-cancerous nasopharyngeal epithelial tissues was detected by immunohistochemistry. The expression of p-Mnk1 and p-eIF4E protein was detected by immunohistochemistry using specific antibodies as described in the section of materials and methods. Strong positive expression of p-Mnk1 was found in nuclei of NPC (Fig. 1A, 20×, IHC, AEC staining). Low and weak nuclear positive expression of p-Mnk1 was showed in the non-cancerous nasopharyngeal epithelial cells (Fig. 1B, 20×, IHC, AEC staining). Strong positive expression of p-eIF4E in the cytoplasm of NPC (Fig. 1C, 20×, IHC, AEC staining). Weak positive expression of p-eIF4E in the cytoplasm of the non-cancerous nasopharyngeal epithelial cells (Fig. 1D, 20×, IHC, AEC staining).

We examine the positive expression and cellular location of p-Mnk1 and p-eIF4E in NPC and non-cancerous nasopharyngeal epithelial cells by IHC. Strong positive expression of p-Mnk1 protein (Fig. 1A) was identified in the nuclei of NPC and low positive staining of p-Mnk1 protein was located in the nuclei of non-cancerous nasopharyngeal epithelial cells (Fig. 1B). Strong positive staining of p-eIF4E was found in the cytoplasm of NPC (Fig. 1C) and weak positive expression of p-eIF4E was showed in the cytoplasm of non-cancerous nasopharyngeal epithelial cells (Fig. 1D). The positive percentage of p-Mnk1 and p-eIF4E expression in the NPC and non-cancerous nasopharyngeal epithelium was 83.5% (227/272), 75.4% (205/272), 40.0% (34/85) and 32.9% (28/85), respectively. There were significantly higher expression of p-Mnk1 and p-eIF4E protein in NPC compared to the control of non-cancerous nasopharyngeal epithelium (P<0.001, P<0.001, respectively).

We also analyzed the associations between the p-Mnk1 and p-eIF4E expression and clinicopathological features of NPC patient including age, gender, histological type, clinical stages, cervical lymph node metastasis, and survival status by univariate Chi-Square Test. Data shown in Table 1 indicated that NPC patients with cervical lymph node metastasis presented higher expression of p-eIF4E (P = 0.035) and p-Mnk1 (P = 0.001) than those without lymph node metastasis. The results also indicated a strong negative correlation between positive expression of p-eIF4E (p = 0.013) and p-Mnk1 (p<0.001) and the survival status of NPC patients. However, there was no significant correlation observed between expression of p-eIF4E and p-Mnk1 and the clinical stages of NPC. Also, no significant correlation was found between p-Mnk1 and p-eIF4E protein expression and gender, age, histological classification and TNM stages of NPC patients (Table 1).

**Table 1:** Association between expression of p-Mnk1 and p-eIF4E protein and clinicopathological features of NPC (n = 272).

Characteristics n	p-eIF4E+ (%) ^_^ (%) *P-value*	p-Mnk1+ (%) ^_^ (%) *P-value*
**Age(yr)**		
≤40 (n = 88)	66(75.0) 22(25.0) NS	69 (78.4) 19 (21.6) NS
>40 (n = 184)	139 (75.5) 45 (24.5)	158 (85.9) 26(14.1)
**Gender**		
Female (n = 65)Male (n = 207)	46 (70.8) 19(29.2)159 (76.8) 48(23.2) NS	56(86.2) 9(13.8)171(82.6) 36 (17.4) NS
**Histological type**		
DNKC (n = 25)	20(80.0) 5(20.0) NS	21 (84.0) 4 (16.0) NS
UDC (n = 247)	185(74.9) 62(25.1)	206(83.4) 41(16.6)
**Clinical stages**		
I and II (n = 94)	71 (75.5) 23(24.5) NS	74 (78.8) 20(21.2) NS
III and IV (n = 178)	134 (75.3) 44(24.7)	153(86.0) 25(14.0)
**LN status**		
LNM (n = 194)	153(78.9) 41 (21.1) 0.035	169 (87.1) 25 (12.9) 0.01
No LNM (n = 78)**Survival status**	52 (66.7) 26(33.3)	58(74.4) 20(25.6)
Alive (n = 177)	125 (70.6) 52 (29.4) 0.013	135(76.3) 42(23.7) 0.000
Dead (n = 95)	80 (84.2) 15(15.8)	92 (96.8) 3(3.2)

Abbreviations: NS, non significant; DNKC: Differentiated non-keratinized carcinoma, UDC: Undifferentiated carcinoma; LN, lymph node; LN, LNM, lymph node metastasis.

### The Comparison of Expression of p-Mnk1 and p-eIF4E in Primary NPC and the Matched Metastatic or Relapsed NPC

**Figure 2:**
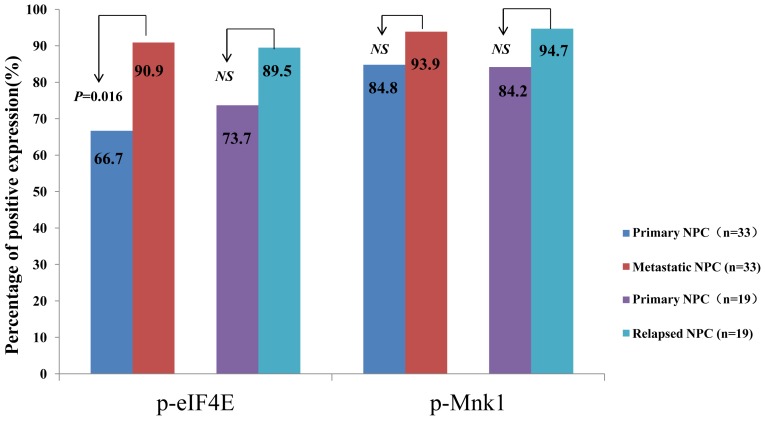
The comparison of expression of p-Mnk1 and p-eIF4E in primary NPC and the matched metastatic or relapsed NPC. We then compared the expression of p-eIF4E and p-Mnk1 in the 52 cases of primary NPC and the matched metastatic or relapsed cancer. The positive expression of p-eIF4E in the primary NPC was significantly lower than that in the matched metastatic cancer (p = 0.016). However, there was no statistically significant difference between expression of p-Mnk1 in the primary NPC and that of the matched metastatic cancer (P>0.05). Also, the positive expression of p-Mnk1 and p-eIF4E was not significant difference between the primary NPC and the matched relapsed cancer.

We then compared the expression of p-Mnk1 and p-eIF4E in the matched primary and metastatic NPC, and in the matched primary and relapsed NPC. As shown in Figure 2, the positive percentage of p-eIF4E expression in the primary NPC tissue was significantly lower than that in the matched metastatic cancer (p = 0.016). Although the positive percentage of p-Mnk1 expression in the primary NPC was also higher than their matched metastatic cancer, the difference did not reach the statistical significance (P>0.05). Also, there was no significant difference in the positive percentage of p-Mnk1 and p-eIF4E expression between the matched primary and relapsed NPC despite the higher expression of both proteins in the matched relapsed NPC (94.7% vs 84.2% for p-Mnk1 and 89.5% vs 73.7% for p-eIF4E).

### The Pairwise Association between Expression of p-Mnk1 and p-eIF4E Protein in NPC

We further evaluated the association between the expression of p-Mnk1 and p-eIF4E in the total 272 NPC patients using the Spearman’s rank correlation test. Data shown in Table 2 indicated a strong positive correlation between the expression of p-Mnk1 and p-eIF4E (r = 0.343, P<0.001).

**Table 2:** The pairwise association between expression of p-Mnk1 and p-eIF4E protein in the 272 cases of NPC.

	p-Mnk1		
	Positive (%)	Negative (%)	*P-value*
**p-eIF4E**			
Positive (%)	186 (68.4)	19(7.0)	0.000
Negative (%)	41(15.1)	26 (9.5)	(r = 0.343)

### Impact of Expressions of p-Mnk1 and p-eIF4E Protein on the Prognosis of NPC

To further examine the impact of expressions of p-Mnk1 and p-eIF4E on the survival of NPC, we employed the Kaplan-Meier analysis to plot the survival curve of all 272 NPC patients, and statistical significance was assessed using the log-rank test. At the time of analysis, the number of NPC specific deaths was 95 cases (34.9%).

**Figure 3:**
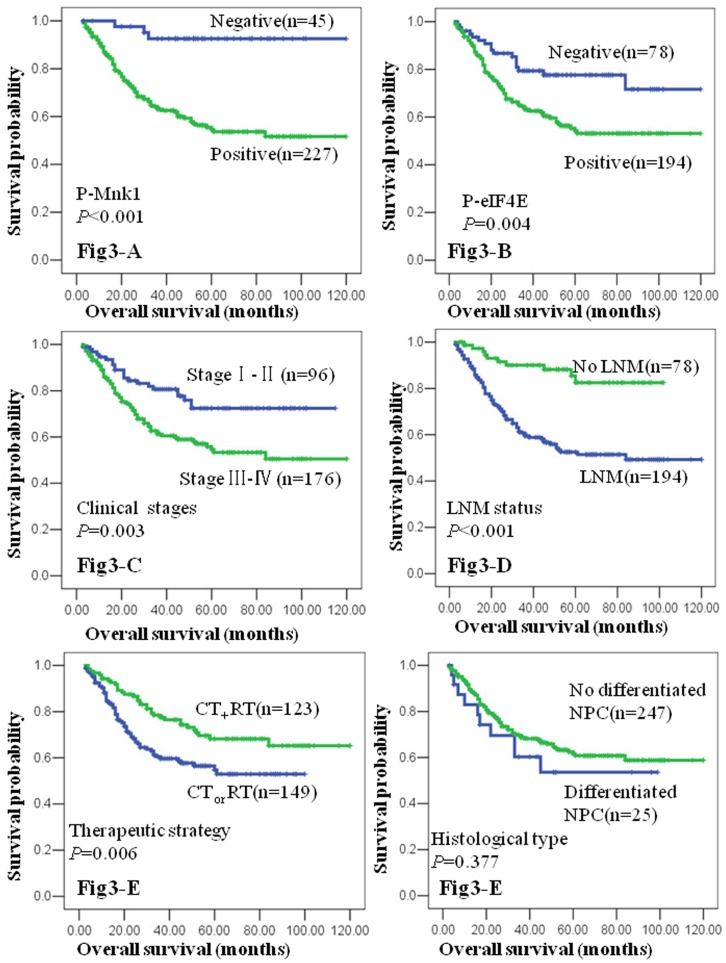
Kaplan-Meier overall survival curves of NPC patients with expression of p-Mnk1 and p-eIF4E protein and different clinicopathological characteristics. Kaplan-Meier analysis to plot the survival curve of all 272 NPC patients with expression of p-Mnk1 and p-eIF4E and different clinicopathological characteristics and statistical significance was assessed using the log-rank test. Fig. 3A. Kaplan-Meier curves showed worse overall survival for p-Mnk1–positive patients compared with p-Mnk1–negative patients (P<0.001, two sided). Fig. 3B. Kaplan-Meier curves showed worse survival for p-eIF4E–positive patients compared with p-eIF4E–negative patients (P = 0.004, two sided). Fig. 3C. NPC patients with clinical stage III and IV were significantly related to poor prognosis compared to those patients with clinical stage I and II (P = 0.003, two sided). Fig. 3D. NPC patients with lymph node metastasis were significantly related to poor prognosis compared to those patients without lymph node metastasis (P<0.001, two sided). Fig. 3E NPC patients with combination chemotherapy and radiotherapy were significantly related to good prognosis compared to patients with chemotherapy and radiotherapy alone (P = 0.006, two sided). Fig. 3F. Histological types of NPC patients were no significantly related to their prognosis (P>0.05, two sided).

Figure 3 illustrates the Kaplan-Meier survival plots for NPC patients with different expression levels of p-Mnk1 (Figure 3A) and p-eIF4E (Figure 3B) protein. Univariate survival (log-rank test) analysis showed that the overall survival rate for NPC patients with positive expression of p-Mnk1 and p-eIF4E was much lower than that for NPC with negative p-Mnk1 and p-eIF4E expression (p<0.001 and p = 0.004, respectively). We also plotted the survival curve for NPC patients with the conventional prognostic parameters, including clinical stages, cervical lymph nodal status, therapy strategy and the histological type. As shown in Figure 3 C-F, lower clinical stages, absence of cervical lymph node metastasis, and combined therapeutic strategy had a positive impact on the overall NPC survival. The NPC patients with locally advanced stage NPC (clinical stage III and IV) and cervical lymph node metastasis had lower overall survival than that patients with early stage NPC (clinical stage I and II) and without lymph node metastasis (p = 0.003, Figure 3C, and p<0.001, Figure 3D, respectively). NPC patients treated with combined chemotherapy and radiotherapy had significantly high overall survival rate than those of radiotherapy alone or chemotherapy alone (p = 0.006, Figure 3E). However, there was no significant difference in the survival between differentiated non-keratinized carcinoma, and undifferentiated carcinoma (P>0.05, Figure 3F). Also, there was no statistically significant difference in overall survival rates among gender and age group by log-rank test analysis (P>0.05, respectively).

To determine whether p-Mnk1 and p-eIF4E were the independent prognostic parameters for NPC, a multivariate Cox proportional hazard regression analysis was carried out to further evaluate the expression of p-Mnk1 and p-eIF4E protein as the prognostic factors. As summarized the Table 3, the positive expression of p-Mnk1 and p-eIF4E protein, clinical stages, cervical lymph node metastasis (LNM), treatment strategy for NPC patients (radiation therapy alone or chemotherapy alone, and combination of radiotherapy and chemotherapy) were significantly correlated with overall survival of NPC patients (p = 0.05, p = 0.001, p<0.001, p = 0.001 and p = 0.004, respectively). Again, no effect was detected with age, gender and histological type of NPC (p>0.05 for all, Table 3). These results of multivariate analysis proved that high expression of p-Mnk1 and p-eIF4E in NPC was independent prognostic factor of overall survival regardless of LNM, clinical stages and combination radiotherapy and chemotherapy, histological type, age and gender.

**Table 3:** Summary of multivariate analysis of Cox proportional hazards model for overall survival in 272 cases of NPC patients.

Parameter		*P-value*	HR	95%CI
**Gender**				
Female vs male		0.117	1.550	0.896–2.679
**Age(yr)**				
>40 vs ≤40		0.343	1.262	0.780–2.040
**Histological type**				
DNKC vs UDC		0.190	0.638	0.326–1.250
**Treatment strategy**				
Combination RT-CT vs RT or CT		0.004	0.517	0.331–0.806
**Clinical stages**				
I-II vs III-IV		0.001	1.647	1.239–2.190
**LN status**				
LNM vs No LNM		0.000	3.686	1.890–7.188
**p-eIF4E expression**				
Positive vs negative		0.050	1.722	1.000–2.966
**p-Mnk1 expression**				
Positive vs negative		0.001	6.755	2.219–21.429

Abbreviations: DNKC: Differentiated non-keratinized carcinoma, UDC: Undifferentiated carcinoma; LN, lymph node; LNM, lymph node metastasis. Chemotherapy: CT, Radiotherapy: RT.

## Discussion

Increasing evidences have shown that Mnks and eIF4E play important roles in the pathogenesis and prognosis of many tumors. Mnks is an upstream kinase of eIF4E which its phosphorylation depends on kinase Mnks activity. The phosphorylation of eIF4E promotes proliferation and survival rate of tumor cell and are critical for malignant transformation and cancer progression [24]–[27], [34], [38]. Inhibition of eIF4E phosphorylation reduces cell growth and proliferation in primary central nervous system lymphoma cells [24]. Mnk1 overexpression is sufficient to confer resistance to trastuzumab in cells that are previously sensitive to the treatment. The phosphorylated Mnk1 is required for the ability of Mnk1 to mediate resistance to trastuzumab. Mnk1 inhibitor leads to reduced cyclin D1 expression and causes inhibition of cell proliferation and cell death in human brain malignant lymphoma cell line [24], [34]–[35]. The significantly abnormal over-expression of p-eIF4E protein is found in a number of tumors including non-small cell lung cancer, breast cancer, gastric cancer, colon cancer, prostate cancer, penile squamous cell carcinoma, head and neck cancer and primary central nervous system lymphoma [24]–[25], [27]–[32]. Previously we reported that phosphorylated eIF4E is elevated in human head and neck squamous cell cancer [31]. In the present study, our results showed there was a high positive expression of p-Mnk1 and p-eIF4E in NPC, but p-Mnk1 and p-eIF4E were significantly low and weak positive expression in the non-cancerous nasopharyngeal epithelia tissues. In this study, we further found that expression of p-Mnk1 and p-eIF4E was significantly positive correlation in NPC. It suggests that the AKT/mTOR and MAPK/MNKs signal pathway augment to promote the development and progression of NPC.

Invasion and metastasis are the basic biological character which is related to recurrence and also effect on NPC patients’ survival [3]–[4], [36], [39]. The invasion and metastasis had correlation with vascular endothelial growth factor (VEGF), cell adhesion and cellular degradation, and positive correlation with the protein of invasion and metastasis. The induction of VEGF protein by Akt is associated with increased phosphorylation and thus activation of p70S6K and eIF4E-binding protein 1, leading to increased VEGF translation [37]. Our results indicated that expression of p-Mnk1 and p-eIF4E protein in the NPC patients with cervical lymph node metastasis was significant higher than those without lymph node metastasis. There was significantly negatively association between positive expression of p-Mnk1 and p-eIF4E and survival status of NPC patients. Interestingly, in this study, there was significant higher expression of p-eIF4E in metastatic NPC than that in the matched primary cancer. These results suggest that high expression of p-eIF4E and p-Mnk1 maybe play a critical role in promoting invasion and metastasis and relate with the poor progression of NPC patients. However, in our study, expression of p-Mnk1 in primary NPC was no significant difference compared with their matched metastatic/relapsed NPC. These results suggested that p-Mnk1 maybe the equal functions in different clinical stages of NPC. These data were from limited samples. We think the reason is further studies need to be done to investigate it with much samples.

There are many factors that are related with NPC prognosis [1]–[2], [36], [39]. Enhanced eIF4E phosphorylation has been observed in various solid tumors and lymphomas, and p-eIF4E overexpression is correlated with poor prognosis or recurrence, metastases in human tumors [10], [13], [24], [27]–[31]. Our results showed that the NPC patients with positive expression of p-Mnk1 and p-eIF4E had an obvious shorter survival time than these patients with negative staining of p-Mnk1 and p-eIF4E. The higher incidence of cervical lymph node metastasis without typical early clinical features and higher rate of loco-regional relapse compared with other head and neck cancers are the main factors on prognosis of NPC. Among a number of classical prognostic indicators for NPC, cervical lymph node metastases is the main one, besides other biological parameters [14], [39]. Fortunately, the early stage NPC patients are usually radio-and chemo-therapy sensitive [5]–[6]. Radiotherapy is the primary treatment modality, and using radiation therapy in combination with chemotherapy is recommended for the treatment of locoregionally advanced tumors. Compared with radiotherapy alone, concomitant chemoradiotherapy represents one of the most recent advances in the treatment of NPC patients and improved the treatment outcome of patients with NPC [5]–[7], [39]. Our results showed that positive expression of p-Mnk1 and p-eIF4E protein, cervical lymph node metastasis, clinical stages, and combination of radiotherapy and chemotherapy were also significantly correlated with overall survival rates of NPC patients by univariate analysis. Multivariate analysis proved that the p-Mnk1 and p-eIF4E positive expression were the independent factors of prognosis for NPC exclude different therapy strategy, clinical stages and cervical lymph node metastasis. Therefore, over-expression of p-Mnk1 and p-eIF4E acts as novel prognostic molecular markers for NPC.

In summary, we have examined the expression of p-Mnk1 and p-eIF4E in NPC and in the non-cancerous nasopharyngeal epithelial specimens, and we further compared their expression between the matched primary and metastatic or relapsed NPC tissues. By analyzing the association of p-Mnk1 and p-eIF4E and clinicopathological characteristics of NPC, we first report that the high expression of p-Mnk1 and p-eIF4E is associated with cervical lymph node metastasis and the poor survival of NPC. The p-Mnk1 and p-eIF4E might be independent prognostic factors of NPC and therefore important therapeutic targets for developing the effective treatment strategies for NPC.
